# Centers for Oceans and Human Health: a unified approach to the challenge of harmful algal blooms

**DOI:** 10.1186/1476-069X-7-S2-S2

**Published:** 2008-11-07

**Authors:** Deana L Erdner, Julianne Dyble, Michael L Parsons, Richard C Stevens, Katherine A Hubbard, Michele L Wrabel, Stephanie K Moore, Kathi A Lefebvre, Donald M Anderson, Paul Bienfang, Robert R Bidigare, Micaela S Parker, Peter Moeller, Larry E Brand, Vera L Trainer

**Affiliations:** 1 University of Texas Marine Science Institute, Port Aransas, TX 78373, USA; 2 NOAA Great Lakes Environmental Research Laboratory, Ann Arbor, MI 48105, USA; 3 Department of Marine and Ecological Sciences, Coastal Watershed Institute, Florida Gulf Coast University, Fort Myers, FL 33965-6565, USA; 4 Department of Medicine (Div. of Medical Genetics), University of Washington, Seattle, WA 98195, USA; 5 Pacific Northwest Center for Human Health and Ocean Studies, School of Oceanography, University of Washington, Seattle, Washington 98195-7940, USA; 6 School of Oceanography, University of Washington, Seattle, Washington 98195-5351, USA; 7 NOAA Northwest Fisheries Science Center, WEst Coast Center for Oceans and Human Health, 2725 Montlake Blvd. E., Seattle, WA 98112, USA; 8 Biology Department, Woods Hole Oceanographic Institution, Woods Hole, MA 02543, USA; 9 Center for Oceans and Human Health, Pacific Research Center for Marine Biomedicine, School of Ocean and Earth Science and Technology, University of Hawaii, Honolulu, HI, 96822, USA; 10 Center for Marine Microbial Ecology and Diversity, University of Hawaii at Manoa, Honolulu, HI 96822-2327, USA; 11 Toxin/Natural Products Chemistry Program, National Ocean Service, NOAA, Hollings Marine Laboratory, Charleston, SC 29412, USA; 12 University of Miami, RSMAS, Miami, FL 33149, USA

## Abstract

**Background:**

Harmful algal blooms (HABs) are one focus of the national research initiatives on Oceans and Human Health (OHH) at NIEHS, NOAA and NSF. All of the OHH Centers, from the east coast to Hawaii, include one or more research projects devoted to studying HAB problems and their relationship to human health. The research shares common goals for understanding, monitoring and predicting HAB events to protect and improve human health: understanding the basic biology of the organisms; identifying how chemistry, hydrography and genetic diversity influence blooms; developing analytical methods and sensors for cells and toxins; understanding health effects of toxin exposure; and developing conceptual, empirical and numerical models of bloom dynamics.

**Results:**

In the past several years, there has been significant progress toward all of the common goals. Several studies have elucidated the effects of environmental conditions and genetic heterogeneity on bloom dynamics. New methods have been developed or implemented for the detection of HAB cells and toxins, including genetic assays for *Pseudo-nitzschia *and *Microcystis*, and a biosensor for domoic acid. There have been advances in predictive models of blooms, most notably for the toxic dinoflagellates *Alexandrium *and *Karenia*. Other work is focused on the future, studying the ways in which climate change may affect HAB incidence, and assessing the threat from emerging HABs and toxins, such as the cyanobacterial neurotoxin β-N-methylamino-L-alanine.

**Conclusion:**

Along the way, many challenges have been encountered that are common to the OHH Centers and also echo those of the wider HAB community. Long-term field data and basic biological information are needed to develop accurate models. Sensor development is hindered by the lack of simple and rapid assays for algal cells and especially toxins. It is also critical to adequately understand the human health effects of HAB toxins. Currently, we understand best the effects of acute toxicity, but almost nothing is known about the effects of chronic, subacute toxin exposure. The OHH initiatives have brought scientists together to work collectively on HAB issues, within and across regions. The successes that have been achieved highlight the value of collaboration and cooperation across disciplines, if we are to continue to advance our understanding of HABs and their relationship to human health.

## Background

For residents of marine and freshwater coastal regions, perhaps one of the most visible manifestations of the interdependence of human and ocean health are the episodic harmful algal blooms (HABs) that affect almost every part of the U.S. coastline. HABs are natural phenomena caused by the proliferation of algae, resulting in damage to the environment and/or risk to the health of humans and aquatic life. Many HAB species produce toxins that are accumulated and passed up the food chain, causing illness or death in humans and other organisms that consume them. Nontoxic organisms can also cause blooms. These so-called "noxious" or "nuisance" bloom species grow to high biomass and cause oxygen depletion, reduction in biodiversity, physical damage, and shading of the benthos. The frequency and severity of HAB events appears to be increasing globally [1-3], which likely reflects both a real increase in HAB events as well as improved monitoring and awareness. Although HABs are often primarily considered natural phenomena, the magnitude and occurrence of some species may be exacerbated by anthropogenic input of nutrients (eutrophication) and perhaps other forms of coastal pollution [3,4]. The influence of climatic and environmental variation on HAB incidence is also an active area of research, as changing global conditions may affect future HAB risk (see below, and [5] in this supplement)

**Figure 1:**
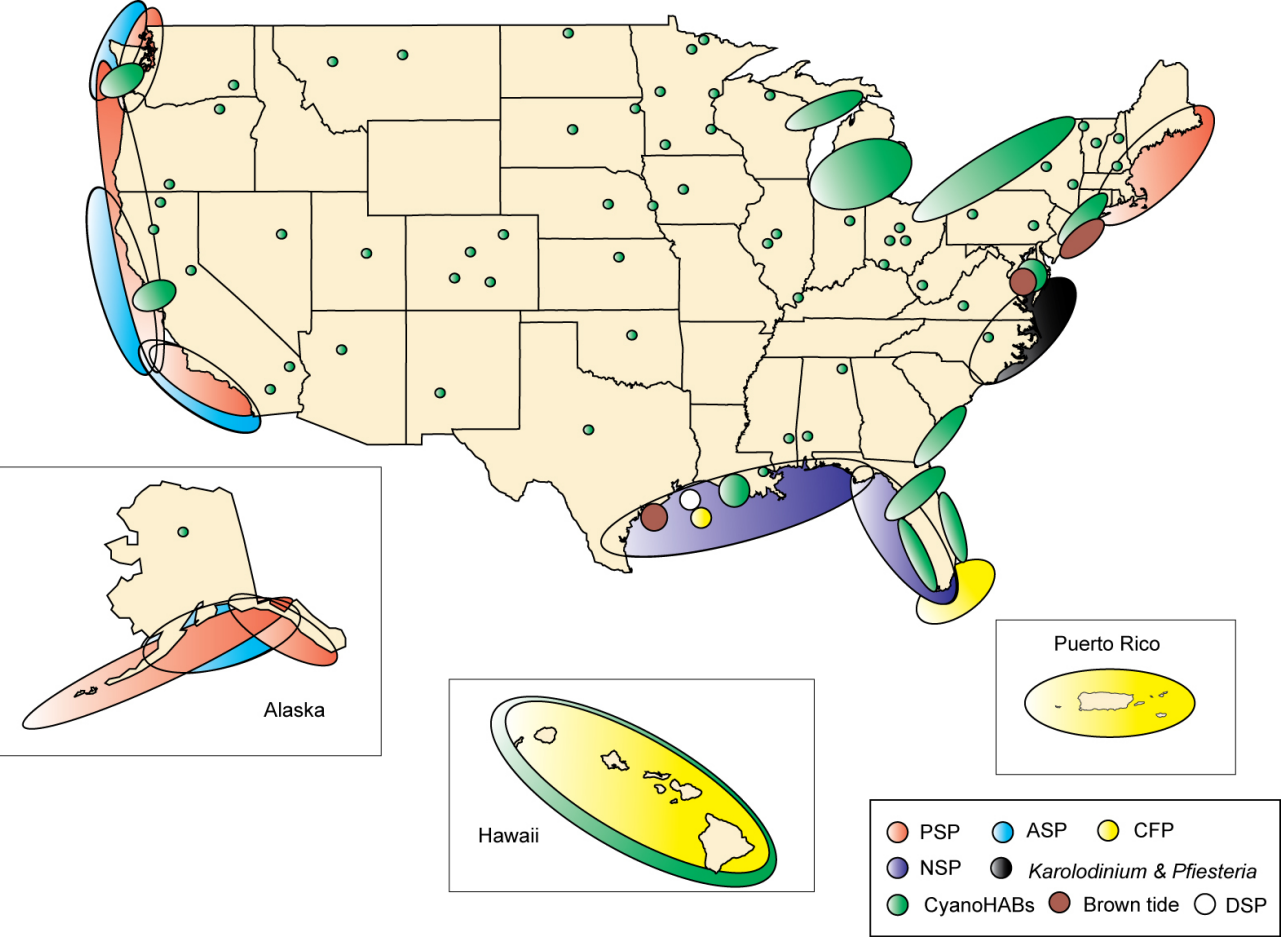
Approximate areas of the U.S. coast affected by various HAB poisoning syndromes and other impacts. Source: U.S. National Office for Harmful Algal Blooms.

HABs are a key focal area of the national research initiatives on Oceans and Human Health (OHH) at NSF/NIEHS and NOAA. All of the OHH Centers, spread from the east coast to Hawaii, include one or more research projects devoted to understanding HAB problems. Collectively, the Centers are engaged in intense study of a variety of major recurrent toxic HAB species in the U.S.: *Alexandrium tamarense, Gambierdiscus *spp., *Karenia brevis, Microcystis *spp., and *Pseudo-nitzschia *spp. (Figure 1). Emerging HAB problems, such as the cyanobacteria responsible for β-N-methylamino-L-alanine poisoning, are also under investigation.

### *Alexandrium tamarense*

Blooms of neurotoxic dinoflagellates of the genus *Alexandrium *can cause outbreaks of paralytic shellfish poisoning (PSP), the most widespread of the HAB-related shellfish poisoning syndromes. PSP is caused by saxitoxins, a family of toxins that bind to sodium channels in nerve and muscle cells, and results from ingestion of shellfish that accumulate toxins from feeding on *Alexandrium *cells. The economic, public health, and ecosystem impacts of PSP outbreaks take a variety of forms and include human intoxications and death from contaminated shellfish or fish, loss of natural and cultured seafood resources, impairment of tourism and recreational activities, alterations of marine trophic structure, and death of marine mammals, fish, and seabirds. The economic impact of *Alexandrium *blooms is substantial; for example, economists estimated that a large bloom in the northeastern U.S. in 2005 cost the seafood industry alone $2.7 million per week in lost revenues, with some estimates suggesting double that amount [6]. Toxic *Alexandrium *species are found worldwide in temperate coastal and estuarine waters. Thousands of miles of U.S. coastline are affected, with recurrent blooms affecting both the northeastern and western coasts of the U.S.

### *Gambierdiscus toxicus*

The toxic dinoflagellate *Gambierdiscus *spp. is the primary source of ciguatoxin, the poison that causes ciguatera fish poisoning (CFP). Humans acquire ciguatoxin (CTX) by eating reef fish that have accumulated the toxin via the marine food web. *Gambierdiscus *dinoflagellates are found in association with various macroalgae in coral reef ecosystems. These dinoflagellates are consumed by herbivorous fish, beginning a process of bioaccumulation, biomagnification, and biomodification in the reef food web, as the herbivores are consumed by carnivores, and, ultimately, by humans. *Gambierdiscus *is found in tropical and subtropical coral reef regions worldwide; in the U.S. it has been reported from Hawaii [7,8], the Florida Keys (e.g. [9]), and the Gulf of Mexico [10]. CFP produces gastrointestinal, neurological, and cardiovascular symptoms, and intoxication does not result in immunity, but rather confers hypersensitivity. Though seldom fatal, CFP symptomatology may include >80 physiological disorders, and prevention is the primary clinical and technical objective. The frequency of CFP worldwide is estimated at 500,000 cases per year [11-13], and records from various Pacific locales suggest that over 80–96% of human fish poisonings are due to CFP. A serious public health threat in the U.S., CFP is estimated to account for >95% of medical costs associated with harmful algal blooms. These toxins may also affect fish directly, altering behavior and possibly compromising immune responses, thereby impacting ecosystem health.

### *Karenia brevis*

The toxic dinoflagellate, *Karenia brevis*, can be found in low concentrations throughout much of the Gulf of Mexico, but episodically forms large blooms [14-16]. These have been observed in the coastal waters of Mexico, Texas, Louisiana, Mississippi, Alabama and western Florida, but they are largest and most frequent along the southwest coast of Florida [15,16]. The blooms can sometimes extend from north Florida to the Florida Keys, and can last from weeks to more than a year. An analysis of the 50-year data record suggests that *K. brevis *has become more abundant during this time [17], though this contention is not universally held among scientists. The highest concentrations of *K. brevis *occur within five kilometers of the shoreline [17], where they have the greatest impact on humans. *K. brevis *produces a suite of neurotoxins called brevetoxins, which can cause neurotoxic shellfish poisoning (NSP), in which nerve cell paralysis results in gastroenteritis, muscle cramps, seizures, paralysis, and other neurological symptoms after the consumption of toxic shellfish [18,19]. Due to vigilant monitoring programs by government agencies, intoxication of humans is now rare. A more widespread effect of *K. brevis *blooms is the release of brevetoxins from the cells, which then form a toxic aerosol that can cause respiratory problems in humans, especially those with asthma [20-22]. This frequently has a major impact on residents and tourists along the southwest coast of Florida, where blooms occur almost annually, resulting in significant financial losses. Brevetoxins can also kill large numbers of fish, which has caused declines in both commercial and recreational fish stocks. Threatened species such as sea turtles, dolphins, and manatees are also killed by *K. brevis *blooms [23,24]. The brevetoxin-related deaths of these animals are particularly significant because of their small population sizes and low reproductive rates.

### *Microcystis*

Cyanobacterial HABs have been observed in the Great Lakes for decades. In the 1960s and 1970s massive blooms of diverse cyanobacteria (*Anabaena*, *Aphanizomenon *and *Microcystis*) occurred during summer when the water column was thermally stratified [25,26], but have since declined due to phosphorus reduction strategies. However, since the mid-1990s there has been a resurgence of cyanobacterial HABs in the Great Lakes and the community is now dominated by *Microcystis*. Large persistent *Microcystis *blooms in the relatively warm and shallow regions of the Great Lakes (including western Lake Erie, Saginaw Bay in Lake Huron, Bay of Quinte in Lake Ontario) have been present during most summers. *Microcystis *produces the hepatotoxin microcystin that is responsible for mortality in livestock, wildlife and birds. Human health effects of exposure to microcystins in drinking water include gastrointestinal illness for acute exposure and increased liver disease and damage and possible tumor promotion for chronic exposure [27]. Recreational exposure to microcystins may result in skin irritation and allergic reactions [28]. This is particularly significant because the Great Lakes provide drinking water to over 40 million U.S. and Canadian citizens and have over 500 beaches that are used for recreational activities. *Microcystis *cells tend to accumulate at the water surface, particularly in calm conditions, resulting in dense surface scums in embayments, against structures such as docks and piers, and in nearshore regions where humans are most likely to come in contact with the water, further increasing the potential impacts on human health.

### *Pseudo-nitzschia*

The diatom genus *Pseudo-nitzschia *contains multiple species known to produce a potent neurotoxin called domoic acid (DA) [29]. The toxin is bioaccumulated in marine shellfish and finfish and subsequently transferred to higher trophic levels [30-33]. Consumption of contaminated seafood by humans results in a neurotoxic illness known as amnesic shellfish poisoning (ASP) [34,35]. The first documented case of ASP occurred in 1987 in Prince Edward Island, Canada, when 3 people died and several others suffered permanent health effects after consuming DA-contaminated mussels [35,36]. Sublethal effects of DA exposure include: vomiting, diarrhea, confusion, disorientation, seizures, and permanent short-term memory loss [36,37]. *Pseudo-nitzschia *is a cosmopolitan genus found in both coastal and open ocean regions from tropical to arctic latitudes. In the U.S., toxic *Pseudo-nitzschia *species have been documented along the west coast [38], in the Chesapeake Bay [39], the Gulf of Mexico [40], and the northeastern U.S. [41]. Blooms of *Pseudo-nitzschia *are prevalent along the west coast of the United States where they have been responsible for closures of shellfish harvests, sometimes for over a year, resulting in the loss of millions of dollars in revenue for the coastal economy. During toxic events, recreational, commercial and tribal subsistence harvest of clams, valued at over $20 million annually [42], is prohibited and public health is threatened.

## Discussion

### A unified approach to the problem of HABs

While the organisms and environments may be diverse, all of the seven Centers employ a similar strategy for the study of HABs. This approach shares a set of five common goals for understanding, monitoring and predicting HAB events in order to protect and improve human health. These goals are: 1) understanding the basic biology of the organisms; 2) understanding how chemistry, hydrography and genetic diversity influence blooms; 3) developing analytical methods and sensors for cells and toxins; 4) understanding health effects of toxin exposure; and 5) developing and validating conceptual, empirical and numerical models of bloom dynamics for predictive purposes. For all of the Centers, the ultimate goal is to develop better methods and technologies for monitoring and predicting HAB events. The ability to detect cells and toxins at high spatial and temporal resolution and to project that information into the future using predictive models will provide the greatest protection to public health. However, we also need to have a better understanding of the organisms and their ecology if we are to build effective sensors and predictive models.

HAB research at the Oceans and Human Health Centers is part of a large and active research community that includes scientists from around the world. Center activities build upon and complement years of research on all aspects of HAB problems. A strength of the OHH Centers is that they bring together scientists from diverse fields, greatly facilitating collaboration and cooperation across scientific disciplines. The ability to tap the combined expertise within and between Centers has made it easy to approach HAB problems "from all angles," and the successes that have been achieved highlight the value of this interdisciplinary approach to the study of HABs.

### Basic biology of HAB organisms

In comparison to many other organisms, especially model systems, relatively little is known about the basic biology of HAB species. One of the biggest gaps is the lack of information about the biochemical pathways for toxin synthesis, especially in eukaryotic HAB species. Several genera of cyanobacteria produce different toxins, and researchers have completely described the multi-gene systems for the synthesis of microcystins [43] and cylindrospermopsin [44]. A saxitoxin gene cluster has been recently identified in cyanobacteria [45] although the genes for saxitoxin synthesis in dinoflagellates remain elusive. Similarly, none of the genes involved in the synthesis of ciguatoxins, brevetoxins, or domoic acid have been conclusively identified. A set of polyketide synthase genes that may direct the synthesis of the cytotoxic amphidinolides has been identified from the dinoflagellate *Amphidinium *[46]; knowledge of their sequence and function may assist in the elucidation of genes involved in the synthesis of other dinoflagellate polyketide toxins, such as brevetoxins or ciguatoxins. The lack of information for eukaryotic HABs may also soon change as a result of efforts by the broader HAB research community to develop genomic resources for HAB species. Scientists have generated expressed sequence tag (EST) collections for the toxic dinoflagellates *Karenia brevis *and *Alexandrium tamarense *[47,48] and used the data to construct microarrays for gene expression profiling [49]. HABs have also finally entered the genomics age with the recent funding of genome sequences for the harmful pelagophyte *Aureococcus anophagefferens *and the toxic diatom *Pseudo-nitzschia multiseries*. These sequencing efforts should greatly advance overall knowledge of the biology of HAB species.

While we lack in-depth genetic information for many HAB species, we do have relatively good information on their physiology, due to laboratory studies that have investigated the response of cultured isolates to environmental conditions. However, we are just beginning to investigate how these organisms function as part of the complex communities that exist in natural waters. This can include multiple species or strains of the same organism, or multi-species assemblages. For example, researchers are beginning to unravel the complex relationship between toxic diatoms and their associated bacteria. Previous studies have shown that not all *Pseudo-nitzschia *species produce domoic acid [50-52], and cells that can produce DA have a wide variability in toxin production depending on such factors as environmental conditions [53], nutrient stress [54,55], growth phase, and bacteria coexisting with *Pseudo-nitzschia *[56,57]. Other researchers have also demonstrated higher toxin production by *Pseudo-nitzschia *in the presence of bacteria [56], with greater toxin enhancement upon introduction of bacteria isolated from a different geographic location than the *Pseudo-nitzschia *cell [57]. Specific relationships have been described between bacteria and other HAB-forming species [58-60]. Literature related to *Pseudo-nitzschia *has focused on several strains of one species, *Pseudo-nitzschia multiseries*.

Center researchers have built upon this work by examining bacterial assemblages associated with 18 *Pseudo-nitzschia *strains, representing 6 species, using a DNA fingerprinting technique called ARISA (Automated Ribosomal Intergenic Spacer Analysis). ARISA amplifies the intergenic spacer region located between 16S and 23S ribosomal RNA (rRNA) genes in bacteria [61]. This method has been used to relate bacterial fingerprints to distinct ecosystem features [62], seasonal changes [63], and phytoplankton species [64]. Using this method, Center scientists found that *Pseudo-nitzschia *species host significantly different bacterial assemblages. Furthermore, bacterial assemblages associated with species capable of high toxin production differed significantly from those associated with species with low toxin production.

Studies of HAB organisms can be complicated by unusual aspects of their biology and life cycles. For example, while the toxic diatom *Pseudo-nitzschia *has recently been selected for whole genome sequencing, genetic characterization of toxic dinoflagellates such as *Karenia *and *Alexandrium *has been limited to the sequencing of EST libraries. This is primarily because the genome sizes of dinoflagellates are often prohibitively large for full genome sequencing. Estimates of dinoflagellate DNA content range from 3 to 250 pg·cell^-1 ^[65], corresponding to approximately 3000–215,000 Mb (in comparison, the haploid human genome is 3180 Mb). The dinoflagellate *Symbiodinium *has a relatively small genome, ca. 2 pg, and investigatory genome sequencing of this organism is currently underway . Although this genus is not toxic, the genome sequences from this dinoflagellate, along with the development of new, ultra-high throughput sequencing technologies, are encouraging advances that make the prospect of a full genome sequence for a toxic dinoflagellate more likely.

For some HAB species, even controlled laboratory experiments can be a challenge because organisms are often difficult to culture. Many HAB species have complex life cycles that include a sexual phase. For example, clonal *Pseudo-nitzschia *cultures eventually die out if they are not mated with larger cells of the same species. The toxic dinoflagellate *Gambierdiscus *has also proved difficult to maintain in culture; it grows slowly and does not attain high biomass [66-68]. Established cultures tend to become less toxic over time, a phenomenon that frustrates efforts at characterizing the effects of environmental conditions on toxicity. While total toxicity can be monitored using cell-based assays, determination of the different, structurally related toxins requires more costly instrumentation, such as chromatography systems and mass spectrometers. This is the case for many of the other HAB toxins as well. Detecting and quantifying HAB toxins are capabilities that individual laboratories often cannot afford to develop and maintain. This highlights a potential role for Center core facilities in future HAB studies – the development of a centralized source of expertise and technology for toxin detection that would be useful to many.

### Effects of nutrients, hydrography, and genetic diversity on blooms

A necessary step in understanding the formation of HABs is taking what we have learned from laboratory studies of clonal culture isolates and translating that into field populations. In the laboratory, conditions can be controlled so that only one factor varies at a time, whereas natural blooms involve a complex interplay of changing chemical, physical and biological conditions, which vary both in time and space. A common goal of the HAB research at the OHH Centers is to understand the effects of nutrient availability, hydrography, and genetic diversity on HAB formation and development.

In the Great Lakes, the toxicity of *Microcystis *blooms is determined both by environmental factors and the genetic composition of the bloom. Nutrients, light, trace metals and temperature are some of the factors that change bloom toxicity either through direct stimulation of toxin production or through coupling to cell growth and division [69-71]. Statistical modeling conducted as part of a Center collaborative effort has shown that multiple environmental conditions (including annual/episodic events, meteorological patterns, seasonal/intermittent riverine inflows, and annual phosphorus loading) interact with taxon-specific physiological traits to holistically influence late-summer cyanobacterial abundance throughout shallow sections of the Great Lakes [72]. Though clearly environmental factors impact bloom toxicity, microcystin cell quota can vary by as much as 3–4 orders of magnitude between strains, suggesting a strong role for bloom composition in predicting toxicity [73,74]. In western Lake Erie, Center researchers identified multiple genetically distinct populations of *Microcystis *based on sequence analysis of one of the microcystin synthetase genes (*mcyB*), further supporting the role of community composition in bloom toxicity.

In *Pseudo-nitzschia*, genetic diversity is reflected in inter- and intraspecific variability in toxin production, which has complicated the implementation of early warning systems for blooms, largely because *Pseudo-nitzschia *are notoriously difficult to identify. Center research has led to the development of a DNA-based *Pseudo-nitzschia*-specific ARISA for species identification, which is used in tandem with environmental clone libraries for strain identification and to assess the diversity of *Pseudo-nitzschia *communities [75]. Both techniques utilize genus-specific PCR primers with environmental DNA samples to amplify ribosomal internal transcribed spacer (ITS1) sequences, which vary in sequence length and content among species, and in sequence content only among genotypes. The goal is to link changes or differences in species or sub-species diversity with specific environmental factors. This will lead to a better understanding of the environmental conditions that favor the growth, predominance, and toxin production of particular species and/or strains. Most of this Center research is occurring in the coastal and estuarine waters of the Pacific Northwest where *Pseudo-nitzschia *blooms are common. However, global distributions of *Pseudo-nitzschia *types are also being investigated to better evaluate the extent of genetic diversity in this genus. Thus far, the Center project has identified more than 14 putative species of *Pseudo-nitzschia *in Pacific Northwest waters using ARISA, compared to ~7 described by past studies [76]. Some of the identified species and strains are globally distributed, while others appear to be specific to a particular region. For example, the same genotype (i.e. 0% divergence) of *P. delicatissima *was detected in Portugal, Denmark, and throughout the Pacific Northwest [75]. Preliminary results suggest that it shows lower environmental constraints than other species, since it is commonly detected in hydrographically distinct Puget Sound basins and across multiple seasons. In contrast, 24 genotypes of *P. pungens *detected in the Pacific Northwest exhibit distinct distribution patterns and high divergence levels in comparison to other species, suggestive of distinct populations or cryptic species that might co-exist in Pacific Northwest waters.

The presence of multiple populations, strains or species during HAB events is turning out to be the rule, rather than the exception, for many HAB organisms. In addition to the cyanobacteria and diatoms described above, high genetic diversity has also been observed in blooms of the toxic dinoflagellate *Alexandrium fundyense *in the northeastern U.S. Center scientists are using microsatellite markers to analyze the diversity and population structure of *Alexandrium *blooms over consecutive bloom years. Results show that blooms are not genetically "static" – that populations present at the end of a bloom can be distinct from those at the start of a bloom. However, results also show that the starting bloom populations in consecutive years are not significantly different from one another. This apparent paradox challenges our assumptions about the homogeneity of blooms, and about the sources of cells for bloom initiation and propagation. It also highlights the potential of genetic markers; they not only provide information about population structure, but can also help us to understand the sources of toxic cells and to identify mixing of populations.

Center research has also been devoted to understanding the effects of changing conditions (e.g., nutrients, temperature, irradiance) on cell growth and toxicity. For example, Center scientists have established dozens of isolates from *A. fundyense *bloom populations in the northeast and measured their growth rates and toxicity under varying conditions of light and temperature. The heterogeneity observed is remarkable – growth rates vary up to three-fold, and toxicity varies as much as four-fold among isolates grown under the same conditions. These differences may lead to the selection of specific genotypes in natural waters, with a resulting variability in population abundance and toxicity from year to year. The effects of this growth and toxin variability on regional blooms are currently being explored using the Gulf of Maine *Alexandrium *numerical model, also as a Center activity [77].

One of the biggest challenges to understanding toxic blooms is that chemical, biological, and physical conditions can all be changing simultaneously, and not necessarily in concert with one another. It is therefore extremely difficult to determine the proximate cause of changes in a natural bloom. Did the bloom die off because it ran out of nutrients? Because of a parasite? Because the winds changed and forced the bloom offshore? Some combination of these factors? This issue could be addressed through the development of methods for the *in situ *analysis of cell physiology, and the collection of long-term field data sets. If we could determine the status of cells in the field, we could use that knowledge to predict the behavior of blooms. For example, fluorescent, precipitable substrates of alkaline phosphatase have been used to demonstrate phosphorus limitation of cells in the ocean [78]. To date, no such indicators have been successfully used to characterize the growth requirements of HAB species, although their potential continues to spur researchers to seek out molecular markers.

Long-term data sets of field measurements of bloom (and non-bloom) events have the potential to greatly increase our understanding of bloom dynamics. Data from many years or bloom events enable us to assess the factors that change from year to year, or bloom-to-bloom, and correlate those with changes in bloom events. Do blooms only occur in years with above average rainfall? Warm summers? Northeast winds? Unfortunately, long-term synoptic data sets are very difficult to obtain, because a single grant proposal often only allows for a few years of monitoring, and it is challenging to keep a field program funded for the period of time necessary to accumulate a substantial time series. The five-year term of the Centers has provided a great boost to field efforts in many regions. Maintaining these programs will be a challenge, but they provide critical information not just for our conceptual understanding of bloom dynamics, but also for the development and testing of mechanistic models of blooms.

### Development of detection methods and sensors for HAB cells and toxins

Methods for detection of marine algal cells and toxins are one of the primary goals of HAB research at the OHH Centers, but the work presents many challenges. Methods must be sensitive enough for detection well below regulatory limits, while being fast enough to allow for detection of toxicity before human exposure. Current methods for detecting many toxins are based on the exposure of laboratory animals to the suspect toxin, a procedure that is slow and costly, or liquid chromatographic purification of the toxin that lacks sensitivity and requires time-consuming sample preparation. Current research is directed towards developing automated portable algal cell detectors capable of distinguishing toxin-producing strains from innocuous strains and toxin sensors capable of real-time detection with high sensitivity during field operation.

In general, the methods that have been developed for the detection and enumeration of HAB cells are more numerous and diverse than those for HAB toxins. HAB researchers have developed a variety of molecular technologies for cell detection including fluorescent in-situ hybridization (e.g. [79]), sandwich hybridization (e.g. [80]), and quantitative PCR (e.g. [81]). In the case of toxin detection, method development is often complicated by the presence of multiple toxins and multiple toxin congeners. In some cases the toxins differ chemically in only minor ways (though this often does not translate into "minor" activity differences). In other cases toxin-producing organisms synthesize suites of toxic compounds with quite varied molecular structure and functionality. For reliable toxin detection, known toxins require simpler and more accurate methods, while unknown toxins require full molecular structural characterization. To be widely adopted for monitoring and risk management, screening methods must be accurate, easy to use/interpret, readily available, reasonably priced, able to test large numbers of samples quickly, and capable of identifying toxins specifically. Answers to better detection and monitoring will likely be found in processes emphasizing molecular structure rather than bioactivity.

**Figure 2:**
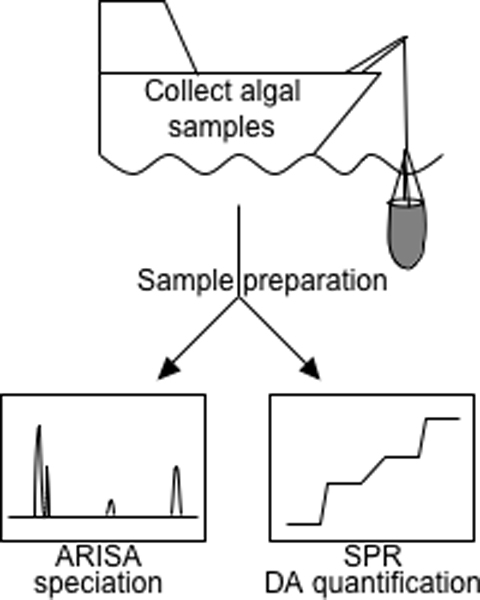
By combining genetic analysis via automated ribosomal intergenic spacer analysis (ARISA) and toxin detection using the surface plasmon resonance (SPR) sensor, it will be possible to identify the species as well as the level of domoic acid associated with a HAB event.

Collaboration has also led to the development of a HAB sensor prototype for the detection of domoic acid, based on a portable surface plasmon resonance (SPR) biosensor. Center scientists raised antibodies raised against domoic acid, which were used to develop competition-based assays for a portable 6-channel SPR system designed by another Center [82]. The current SPR system is capable of detecting up to 24 small molecule, protein, and microbial targets at the same time, which would allow for the detection of several toxins and HAB species simultaneously. Identification of different *Pseudo-nitzschia *species in Puget Sound and Washington coastal waters can be achieved using the ARISA technique described above. Once the sensitivity of both assays is improved, ARISA and SPR biosensor detection could be combined to enable field characterization of HABs (Figure 2).

Molecular methods are also being used to detect and quantify *Microcystis *cells and their toxigenicity. Other researchers have previously identified the genetic pathway for microcystin synthesis, which includes a cluster of 10 genes encoding polyketide synthases and peptide synthetases [43]. Of the 10 genes, PCR-based detection and phylogenetic comparisons are most commonly made based on *mcyA*, *mcyB*, *mcyD *or *mcyE *genes [83-85]. In addition, quantitative real-time PCR methods have been established for the enumeration of toxic *Microcystis *in environmental samples [86,87]. Such methods are being applied by Center scientists in the Great Lakes to determine the distribution of toxic *Microcystis *in the western basin of Lake Erie and Saginaw Bay, the degree of genetic diversity of *Microcystis *populations and the proportion of toxic vs. nontoxic *Microcystis *strains. In other systems dominated by *Microcystis*, strains with and without the genetic potential for microcystin production generally are present [88,89]. Results of Center research suggest that the underlying genetic structure of the population may have a profound effect on the potential toxicity for a given bloom.

Most of the techniques and sensors developed for HAB detection still require a human operator, in a laboratory, which increases the time between sample collection and measurement. Along with cell and toxin data, we need to understand how blooms are driven by and/or respond to chemical and physical properties of the water column, and how toxins produced by some of these organisms propagate through the food web. For these reasons, the study and management of HABs would benefit tremendously from the development of remote, *in situ*, real-time, observing systems capable of detecting specific HAB species and associated toxins.

Direct optical measurements can provide a tractable means of detecting high biomass blooms. HAB scientists are studying toxic red tides that occur in the Gulf of Mexico using an optical sensor fitted onto an inexpensive, unmanned underwater glider that can collect continuous profiles of the water column and its characteristics [90]. However, many HAB species can exert harmful effects at concentrations far below those that lead to visually discolored water and may represent only a small fraction of the total number of cells present in the environment. Since it is not always possible to discriminate harmful from benign organisms based on optical properties alone, other techniques that provide species or even "strain" level resolution are required. Application of molecular probes is one means of accomplishing that goal [91]. When the problem organisms are rare, however, relatively large samples are required (hundreds of mL to liters) and multi-step chemical processing of that material is needed to reveal target species or toxins of interest given the techniques available currently. Therefore, successful application of molecular probe sensing technology for remote detection of a variety of HAB species on autonomous platforms may require instrumentation that both accommodates large sample volumes and provides the means for processing that material prior to delivery to the detector itself. While such systems are emerging [91-93], it is not likely in the short term that they will provide data at rates comparable to those of physico-chemical sensors, such as the combination conductivity, temperature and depth profilers (CTD) that are routinely deployed from research vessels. Consequently, near term application of HAB biosensors fielded on autonomous platforms will likely be tightly integrated with other sensors that trigger an analysis in adaptive fashion based on environmental gradients readily detectable at high frequency and previously identified as favorable for HAB formation.

To date, only a few of the emerging observing systems in marine and freshwater environments include HAB-specific instrumentation, since much of it is still under development. The automated real-time bloom or toxin detection systems emerging now are therefore being operated in parallel with more traditional cell and toxin detection methods to better characterize the robustness of these systems as well as the reliability and accuracy of the data. Despite the challenges that lie ahead, the development of sensors based on molecular, biological and optical techniques is progressing rapidly and holds great promise for application on autonomous platforms for future HAB management and mitigation.

### Understanding the human health effects of toxin exposure

Understanding the health effects of toxin exposure on humans and other animals is necessary if we are to improve prevention efforts and reduce the health risks from HAB events. In the U.S., there are few reported cases of illness from the consumption of shellfish contaminated with known HAB toxins. This is because states have effective monitoring programs that test for toxins in shellfish and, in some cases, the presence of harmful species in coastal waters. However, shellfish consumption is not the only route through which HABs impact human health. The toxins of *K. brevis *can become aerosolized, which results in respiratory irritation and associated illness. *Microcystins *can be ingested through drinking water [94]. We understand the acute toxicity effects associated with the various poisoning syndromes much better than the effects of chronic, subacute toxin exposure. Chronic exposure can result from the regular consumption of seafood with levels of toxin in their tissues below the regulatory limit, and therefore considered safe to eat. Chronic exposure is a serious concern, particularly for sub-populations that rely heavily on seafoods, or susceptible populations such as children and the elderly. This is a critical area that deserves increased attention and would benefit from a truly interdisciplinary approach that considers the ecological, public health and epidemiological aspects of toxin exposure.

Human and animal studies are being conducted to understand the health effects of brevetoxin exposure. Studies of brevetoxin exposure are complicated by the fact that brevetoxin can enter human beings by two major pathways: ingestion of seafood contaminated with the toxins and breathing the aerosol that contains the toxins. Brevetoxin is a lipid-soluble cyclic polyether that is not easily metabolized by organisms and can be passed up the food chain [95]. Because of their lipid-soluble nature, brevetoxins tend to accumulate less in muscle tissue and more in the viscera of organisms [24]. The brevetoxins have no taste or odor, and thus cannot be easily detected by potential human consumers. They are also heat and acid stable, thus cannot be easily destroyed in food preparation [18]. The consumption of bivalve shellfish appears to be the main way in which humans can receive a large dose of brevetoxins in their bodies. However, recent work by HAB scientists has raised concerns that brevetoxins can accumulate in the tissues of finfish, which may represent another potential source of brevetoxin exposure for humans and marine mammals [96]. The risk of brevetoxins to human health is further compounded by an increase in *Karenia *blooms. Center research has shown that average *Karenia *cell concentrations along the west coast of Florida have been increasing over the last 50 years, along with increases in the geographic extent and duration of the blooms [17].

Because *K. brevis *is a relatively delicate unarmored dinoflagellate, the cells can be disrupted by surface wave action, leading to the release of brevetoxin-laden aerosols [97,98]. The degree of human exposure to brevetoxin by this route depends upon a number of factors such as cell concentrations, wind speed and direction, and particle size of the aerosols [99]. Inhalation of aerosolized brevetoxins can lead to eye and respiratory irritation, bronchoconstriction, and a decrease in pulmonary function [18,99]. People affected begin coughing and sneezing, and develop runny noses, watery eyes, irritation in the throat, and have trouble breathing. Humans with asthma are particularly sensitive. A collaborative research project supported by NIEHS and other agencies, and involving Center scientists, has examined the human health consequences of exposure to brevetoxin aerosols. Emergency room admissions for pneumonia, bronchitis, asthma, and other respiratory problems were found to increase substantially during *Karenia *bloom events [100]. While no deaths as a result of brevetoxin alone have ever been recorded [24], the possibility that brevetoxins or their effects could work in synergy with other preexisting health problems to increase death probabilities cannot be discounted. Brevetoxins could also have a number of other less easily detectable and/or longer-term effects, but this potential has not been studied yet.

Center researchers are also examining the effects of sub-acute domoic acid (DA) exposure on gene expression in the vertebrate central nervous system using the zebrafish model system. Zebrafish are currently recognized as one of the most important vertebrate model organisms for gene function and toxicogenomics studies [101]. In addition, the zebrafish genome has been sequenced and large-scale genetic screens have been performed and extensively analyzed [102]. Zebrafish mutations phenocopy many human disorders and results from these studies provide candidate genes and pathways for evaluation of disease in higher organisms. In terms of human health, there are growing concerns regarding toxicity due to chronic DA exposure particularly in Washington coastal Tribal communities that subsistence fish on razor clams, which are known to retain DA for up to a year [32]. The Quinault phrase ta'a Wshi xa'iits'os means "clam hungry," and illustrates the strong cultural reliance on razor clams for food. Currently, clam dig openings are guided by toxin regulatory limits that are set by the Washington State Department of Health (WDOH). However, the concern is that people are likely exposed to low levels of DA (≤ 20 ppm the regulatory limit) on a regular basis (J. Schumacker, Quinault tribe, pers. comm.), with particular concern regarding exposures of infants and children [34]. A joint pilot project between two Centers used a zebrafish microarray to identify a subset of genes that were differentially expressed in the vertebrate central nervous system in response to low level (asymptomatic) DA exposure. Connecting these genes identified in the zebrafish vertebrate model with the physiological processes they regulate will facilitate a more detailed understanding of the human health impacts of low-level chronic exposure to DA.

The primary route of human exposure to microcystins in the Great Lakes is thought to be through drinking water and recreation. In particular, microcystin concentrations in the source waters of Saginaw Bay (Lake Huron), western Lake Erie, Bay of Quinte and Hamilton Harbor (Lake Ontario) have exceeded the recommended threshold of 1 μg/L of microcystin established for finished drinking water. Microcystin concentrations in surface scums have been measured as high as 400 μg/L [103], well exceeding the 20 μg/L recommendation for recreational exposure [103,104]. Another potential exposure route is through fish consumption due to the recreational and commercial importance of fishing in both regions of the Great Lakes and in inland lakes that have significant cyanobacterial HAB concentrations. Center scientists, in collaboration with university partners, have measured microcystin accumulation in the tissues of edible size fish caught in lakes with high cellular microcystin levels. Concentrations of microcystin in the liver would be hazardous to human health, but quantities were significantly lower in muscle tissue [105]. While most populations do not consume fish livers, this route of exposure needs to continue to be evaluated along with increased incidence of cyanobacterial blooms in the recreational and drinking waters, all of which could pose a significant concern for public health in the Great Lakes.

It is sometimes easy to lose sight of the risks that HABs pose to human health, because effective monitoring efforts prevent almost all shellfish poisoning events. The value of HAB research and monitoring is enormous in terms of risk avoidance and mitigation, but it makes epidemiological study of these phenomena extremely difficult. Almost nothing is known about the effects of toxins below these regulatory limits, yet regular consumption of drinking water and seafood may result in chronic low-level toxin exposure. This risk can be particularly pronounced in certain sub-populations, for instance the elderly, or those whose diets rely heavily on seafoods for cultural or subsistence reasons. The risks of low-level toxin exposure during development, either *in utero *or during early childhood, are also unknown. Our understanding of the health risks of HAB toxins, and therefore our ability to mitigate those risks, is incomplete until we know the human health effects associated with both chronic and acute toxin exposures.

### Development of models and prediction capabilities

As mentioned above, one of the common goals of all of the OHH Centers is the development of predictive models that will help us to understand and avoid human health risks from HABs. Dyble et al. 2008 [106] (this supplement issue) provide an in-depth description of the experience of the OHH Centers in model development; here we provide a short overview of some of the successes in the modeling and prediction of HAB events specifically.

**Figure 3:**
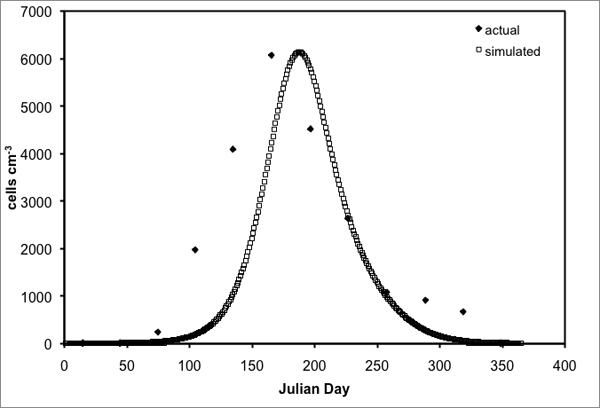
Preliminary results of a model simulating *Gambierdiscus *cell abundance (per cm^3 ^of substrate) versus actual *Gambierdiscus *abundance (monthly-averaged over a three-year period) for the leeward side of the island of Hawaii over a one-year period (365 Julian Days).

Center research generated two of three years of environmental monitoring data on *in situ *temperature, salinity, DIN, phosphate, and light that were incorporated into a series of regression equations to simulate seasonal patterns in these variables for the leeward and windward sides of the island of Hawaii. Cultured isolates were used to determine the physiological response curves of Hawaiian *Gambierdiscus *to different temperatures, nutrient concentrations, light, and salinities. These response curves were coupled with the seasonal patterns of the environmental variables in a *Gambierdiscus *population dynamics model. The model results were then compared to actual *Gambierdiscus *abundance data collected over the three year period at leeward and windward sites on the island of Hawaii to assess its accuracy and realism; preliminary results are shown in Figure 3. In future modeling efforts, the environmental parameters will be subjected to randomized fluctuations (within measured variability) to study the various conditions that can cause a "bloom" of *Gambierdiscus *and a possible, subsequent outbreak of CFP.

Center researchers have also previously developed a coupled three-dimensional physical-biological model to understand, predict, and analyze *Alexandrium *blooms in the northeastern U.S.. *Alexandrium *has a complex life cycle, which includes a resting "cyst" stage that forms at the end of a bloom, and enables the cells to remain dormant until the next spring bloom season begins. The model includes germination of these cysts to begin the bloom, along with the speeds and directions of ocean currents, water temperature, salinity, winds, solar radiation, tides, river runoff, and nutrients to generate a simulation of an *Alexandrium *bloom. Prior simulations have demonstrated that model predictions are quite similar to the actual bloom conditions observed in the field [77]. The model results also indicate that the behavior of the cells, the prevailing wind direction, and the input of fresh water from coastal rivers all play an important role in whether a bloom forms in a given year. Building upon their prior work, Center scientists used the model to perform "hindcast" simulations of a massive *Alexandrium *bloom in 2005 [6] to try to determine which factors were responsible for that unprecedented bloom. Three hypotheses were proposed: high resting cyst abundance, heavy rainfall and runoff, and major storm events ("northeasters") that pushed surface waters and cells to the shore and along the coast. Model conditions were systematically varied to produce the hindcast model simulations of the bloom; results indicate that high abundance of cysts in western GOM was the main cause of the 2005 bloom, although wind forcing was an important regulator. This suggests that monitoring of the regional abundance of cysts may hold the key to interannual forecasts of *A. fundyense *bloom severity. The combination of predictive modeling and the ability to perform hindcast simulations has tremendous power to help us understand the factors that are responsible for *Alexandrium *blooms, and represents a major advancement in HAB management

In Florida, Center research has contributed to a notification system that uses a combination of remote sensing and ground-based monitoring to provide an early warning of *K. brevis *blooms. The west coast of Florida is commonly affected by *K. brevis *blooms, but they are not currently predictable. Florida government agencies routinely monitor the abundance of *K. brevis *and the toxin levels in shellfish in the coastal waters along the west coast. Because of the cost of widespread monitoring, remote sensing is used to target the water sampling. High concentrations of chlorophyll detected by remote sensing are used as potential indicators of *K. brevis *blooms [107]. Targeted water sampling is then used to confirm the satellite data. Once elevated concentrations of *K. brevis *have been confirmed, toxin levels in shellfish are then monitored. Only the west coast is routinely monitored, since other areas are affected so infrequently that monitoring would not be cost effective. Remote sensing however, can track large blooms and monitor any long distance transport of them. For areas not usually hit by *K. brevis *blooms, remote sensing can therefore alert agencies of potential problems so that monitoring can be initiated. Thus, monitoring coupled with remote sensing allows for a more effective use of resources.

Scientists use the satellite data, along with cell counts, meteorological data and oceanographic buoys to monitor bloom movement, for the purpose of predicting future developments. This information is distributed to researchers and other interested partners weekly. Information about *K. brevis *and its effects on humans is routinely presented in newspapers and radio stations along the west coast. Many tourist hotels along the beach also voluntarily provide such information, as tourists are much less aware of Florida's red tide than the local residents. Daily updates on the location of blooms are often posted on beaches, as well as websites, newspapers and radio stations, as *K. brevis *can have a major impact on people's lives and how they may want to change their plans for the day. A toll-free hotline (888-232-8635) is available for people to report any effects they have felt or to ask questions about Florida's red tide. In the future, poison information center and other human health surveillance data as well as real-time monitoring by lifeguards on beaches will be incorporated into the system [108].

The situations in Florida and the northeastern U.S. are not typical – for most HAB organisms, no conceptual or mechanistic models are currently available. One reason for this is the lack of long-term observations and detailed laboratory studies to provide the data to parameterize and constrain the models. Development of the *Alexandrium *model benefited enormously from years of accumulated experimental data on the physiology of the dinoflagellate. This provided information on the behavior of the cysts and cells in response to light, nutrients, and temperature. Numerous research cruises collected data on cell numbers and oceanographic conditions, during bloom and non-bloom years, and on the distribution and abundance of resting cysts in the sediments. All of this information was then coupled with an existing physical model of the region. For many organisms, these basic building blocks for a model are just not available. This underscores the need for long-term observational field programs, and for basic physiological data on the organisms themselves. Our ability to understand and eventually predict HABs depends critically on this type of science.

### Looking forward

The establishment of the OHH Centers has greatly facilitated cooperation between researchers from different scientific disciplines and geographic regions. While Center research addresses the current challenges that HABs present, Center scientists are also cognizant of new, potentially unknown, problems in the future. Two areas that are likely to become more prominent in the near future are emerging HAB species and events, and the effects of global environmental change on HABs (see Moore et al., [5] in this supplement issue).

### Emerging HABs

**Figure 4:**
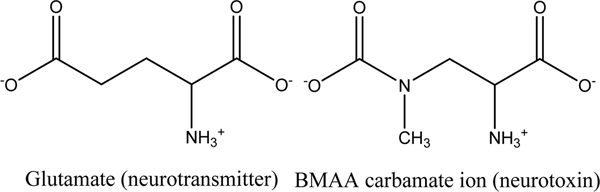
Structural similarities between the neurotransmitter L-glutamate and the neurotoxic carbamate adduct of BMAA.

A previously unknown HAB phenomenon has been identified by Center researchers working with Dr. Paul Cox (Institute for Ethno Medicine) and colleagues. In April 2005, they reported that the neurotoxic glutamate agonist, β-N-methylamino-L-alanine (BMAA), is produced by all known groups of cyanobacteria, including cyanobacterial symbionts and free-living freshwater, marine and terrestrial cyanobacteria [109]; Figure 4). Previous investigations of this neurotoxin in the cyanobacterial symbionts of cycad palm trees in Guam led researchers to hypothesize that BMAA is the putative cause of high-incidence amylotrophic lateral sclerosis/parkinsonism-dementia complex (ALS/PDC) among the Chamorro people. In Guam, human exposure to high quantities of BMAA results from unique components of the traditional Chamorro diet including cycad tortillas, flying foxes, and possibly other feral animals. The ubiquity of cyanobacteria in diverse terrestrial and aquatic environments suggests that ingestion of BMAA may occur through even less esoteric routes, including direct consumption of cyanobacteria or cyanobacterial hosts, bioaccumulation in additional food chains, or exposure to cyanobacteria-contaminated water supplies. Collaboration between two Centers is continuing this line of research by screening a variety of marine and freshwater ecosystems for evidence of BMAA biomagnification and potential human risk.

In addition to previously unknown HAB organisms or toxins, an additional threat comes from the expansion or emergence of known HABs in areas where they have not previously occurred. Historically, *Gambierdiscus *has been reported in the United States only from Hawaii, the Florida Keys, and Puerto Rico, but it has recently been reported in the Gulf of Mexico [10]. In the Gulf, oil platforms serve as colonization sites for *G. toxicus *in areas that are otherwise inhospitable due to unsuitable bottom conditions. The Gulf of Mexico has over 4000 of these producing sites, which provide a nexus for fisherman and potentially toxic fish. Multiple CFP cases have been reported from fish caught near oil platforms [110]. The steel archipelago of the northern and western Gulf of Mexico is a great experiment in providing refuges for tropical benthic species. An ongoing collaboration between investigators in Texas and Center scientists is comparing the CTX produced in these two locations to determine if the ecological source of the toxins is related to differences in molecular weight and/or structure. This information is relevant to the advancement of diagnostic capabilities for ciguatera fish poisoning worldwide.

### HABs and global change

The consequences of projected global change for HABs are generally poorly understood. They are likely to vary for tropical and temperate ecosystems because of differing regional expressions of global change in environmental forcing parameters of HAB dynamics. In the Pacific Northwest, HAB species proliferate primarily during warmer seasons [111], and therefore threaten human health mostly during spring, summer and early fall. Projected increases in water temperature will increase the annual window of favorable growth conditions and may encourage earlier and longer lasting blooms (see Moore et al., [5] in this supplement). Other possible effects of global change will cross regional boundaries, such as range expansions of tropical and temperate HAB species into temperate and northern ecosystems, respectively. For example, CFP caused by the toxic dinoflagellate *Gambierdiscus *occurs primarily between the 35°N and 35°S latitudes. It is believed that any latitudinal extension of warmer water to the north where human populations are greater will similarly increase the incidence of CFP [112]. Such impacts will be most severe in areas that are currently the warmest. Incorporating these kinds of climate change impacts into predictive models and risk assessments of HABs will be paramount to understanding this link between oceans and human health in the future. This can be achieved through a combined approach capitalizing on the wide range of expertise in the OHH centers.

## Conclusion

HAB research at each of the OHH Centers shares a common set of goals that encompasses many of the primary needs and issues for understanding the links between HABs and human health. For all of the Centers, the ultimate goal is the development of better methods and technologies for monitoring and predicting HAB events, so that we can mitigate or eliminate their adverse human health effects. There has been notable progress towards this goal for several HAB organisms, for example the development of toxin sensors for domoic acid and the forecasting and hindcasting of *Alexandrium *blooms. However, considerable challenges remain for all of the HAB problems being studied. Long-term field data and basic knowledge of the effects of nutrient availability, hydrography, and genetic diversity on HAB formation and development are necessary if we are to develop accurate conceptual, empirical and numerical models. Sensor development calls for simple and rapid assays for algal cells and especially toxins. It is also critical for us to adequately understand the human health effects of HAB toxins; we best understand the acute toxicity effects, but almost nothing is known about the effects of chronic, subacute toxin exposure. At the same time that we are working towards filling the current gaps in our knowledge of HABs, we must also be aware of future challenges that we may face. Changing climate conditions may affect the range, frequency and severity of HAB events. New HABs are likely to emerge, as in the case of the cyanobacterial neurotoxin BMAA. The OHH initiatives have brought scientists together to work collectively on a number of HAB problems, both within and across regions. The successes that have been achieved highlight the value of collaboration and cooperation across disciplines to the study of HABs.

## Competing interests

The authors declare that they have no competing interests.

## Authors' contributions

All authors contributed to drafting the manuscript and revising it critically for intellectual content. All authors read and approved the final manuscript.

## References

[B1] Anderson DM, Okaichi, Anderson DM (1989). Toxic algal blooms and red tides: a global perspective. Red tides: biology, environmental science, and toxicology.

[B2] Hallegraeff GM (1993). A review of harmful algal blooms and their apparent global increase. Phycologia.

[B3] Smayda T, Graneli E, Sundstrom B, Edler L, Anderson DM (1990). Novel and nuisance phytoplankton blooms in the sea: Evidence for a global epidemic. Toxic Marine Phytoplankton.

[B4] Anderson DM, Glibert PM, Burkholder JM (2002). Harmful algal blooms and eutrophication: Nutrient sources, composition and consequences. Estuaries.

[B5] Moore SK, Trainer VL, Mantua NJ, Parker MS, Laws EA, Backer LA, Fleming LE (2008). Centers for oceans and human health: Climate variability, climate change and harmful algal blooms. Environmental Health.

[B6] Anderson DM, Keafer DJ, McGillicuddy MJ, Mickelson KE, Keay PS, Libby JP, Manning CA, Whittaker JM, Hickey RH, Lynch DR, Smith KY (2005). Initial observations of the 2005 *Alexandrium fundyense *bloom in southern New England: General patterns and mechanisms. Deep Sea Research II.

[B7] Shimizu Y, Shimizu H, Scheuer PJ, Hokama Y, Oyama M, Miyahara JT (1982). *Gambierdiscus toxicus*, a ciguatera-causing dinoflagellate from Hawaii. Bulletin of the Japanese Society of Scientific Fisheries.

[B8] Taylor FJR, Taylor DL, Seliger HH (1979). A description of the benthic dinoflagellate associated with maitotoxin and ciguatoxin, including observations on Hawaiian material. Toxic Dinoflagellate Blooms.

[B9] Bergmann JS, Alam M (1981). On the toxicity of the ciguatera producing dinoflagellate, *Gambierdiscus toxicus*, isolated from the Florida Keys, USA. Journal of Environmental Science and Health Part A Environmental Science and Engineering.

[B10] Villareal TA, Hanson S, Qualia S, Jester ELE, Granade HR, Dickey RW (2007). Petroleum production platforms as sites for the expansion of ciguatera in the northwestern Gulf of Mexico. Harmful Algae.

[B11] Lehane L, Lewis RJ (2000). Ciguatera: recent advances but the risk remains. International Journal of Food Microbiology.

[B12] Pearn J (2001). Neurology of ciguatera. Journal of Neurology, Neurosurgery and Psychiatry.

[B13] Quod JP, Turquet J (1996). Ciguatera in Reunion Island (SW Indian Ocean): Epidemiology and clinical patterns. Toxicon.

[B14] Geesey M, Tester PA, Smayda TJ, Shimizu Y (1993). *Gymnodinium breve*: ubiquitous in Gulf of Mexico waters?. Toxic Phytoplankton Blooms in the Sea.

[B15] Kusek KM, Vargo G, Steidinger K (1999). *Gymnodinium breve *in the field, in the lab, and in the newspaper-a scientific and journalistic analysis of Florida red tides. Contributions in Marine Science.

[B16] Steidinger KA, Vargo GA, Tester PA, Thomas CR, Anderson DM, Cembella AD, Hallegraeff GM (1998). Bloom dynamics and physiology of *Gymnodinium breve *with emphasis on the Gulf of Mexico. Physiological Ecology of Harmful Algal Blooms.

[B17] Brand LE, Compton A (2007). Long-term increase in *Karenia brevis *abundance along the southwest Florida coast. Harmful Algae.

[B18] Kirkpatrick B, Fleming LE, Squicciarini D, Backer LC, Clark R, Abraham W, Benson J, Cheng YS, Johnson D, Pierce R (2004). Literature review of Florida red tide: implications for human health effects. Harmful Algae.

[B19] Van Dolah FM, Roelke D, Greene RM (2001). Health and ecological impacts of harmful algal blooms: Risk assessment needs. Human and Ecological Risk Assessment.

[B20] Fleming LE, Backer LC, Baden DG (2005). Overview of aerosolized Florida red tide toxins: Exposures and effects. Environmental Health Perspectives.

[B21] Fleming LE, Kirkpatrick B, Backer LC, Bean JA, Wanner A, Reich A, Zaias J, Cheng YS, Pierce R, Naar J (2007). Aerosolized Red Tide Toxins (Brevetoxins) and Asthma. Chest.

[B22] Pierce RH, Henry MS, Blum PC, Hamel SL, Kirkpatrick B, Cheng YS, Zhou Y, Irvin CM, Naar J, Weidner A (2005). Brevetoxin composition in water and marine aerosol along a Florida beach: Assessing potential human exposure to marine biotoxins. Harmful Algae.

[B23] Landsberg J, Steidinger K, Reguera B, Blanco J, Fernández ML, Wyatt T (1998). A historical review of *Gymnodinium breve *red tides implicated in mass mortalities of the manatee (*Trichechus manatus *Latrirostris) in Florida, USA. Harmful Algae.

[B24] Landsberg LH (2002). The effects of harmful algal blooms on aquatic organisms. Reviews in Fisheries Science.

[B25] Bierman VJ, Dolan DM, Kasprzyk R, Clark FL (1984). Retrospective analysis of the response of Saginaw Bay, Lake Huron, to reductions in phosphorus loadings. Environmental Science and Technology.

[B26] Brittain SM, Wang J, Babcock-Jackson L, Carmichael WW, Rinehart KL, Culver DA (2000). Isolation and characterization of microcystins, cyclic heptapeptide hepatotoxins from a Lake Erie strain of *Microcystis aeruginosa*. Journal of Great Lakes Research.

[B27] Falconer IR, Humpage A (1996). Tumour promotion by cyanobacterial toxins. Phycologia.

[B28] Codd GA, Bell SG, Kaya K, Ward CJ, Beattie KA, Metcalf JS (1999). Cyanobacterial toxins, exposure routes and human health. European Journal of Phycology.

[B29] Bates SS (2000). Domoic acid-producing diatoms: Another genus added!. Journal of Phycology.

[B30] Lefebvre KA, Silver MW, Coale SL, Tjeerdema RS (2002). Domoic acid in planktivorous fish in relation to toxic *Pseudo-nitzschia *cell densities. Marine Biology.

[B31] Scholin CA, Gulland F, Doucette GJ, Benson S, Busman M, Chavez FP, Cordaro J, DeLong R, De Vogelaere A, Harvey J (2000). Mortality of sea lions along the central California coast linked to a toxic diatom bloom. Nature.

[B32] Wekell JC, Gauglitz EJ, Barnett HJ, Hatfield CL, Simons D, Ayres D (1994). Occurrence of domoic acid in Washington state razor clams (*Siliqua patula*) during 1991–1993. Natural Toxins.

[B33] Wright JLC, Boyd RK, de Freitas ASW, Falk M, Foxall RA, Jamieson WD, Laycock MV, McCulloch AW, McInnes AG, Odense P (1989). Identification of domoic acid, a neuroexcitatory amino acid, in toxic mussels from eastern Prince Edward Island. Canadian Journal of Chemistry.

[B34] Grattan IM, Lesoing M, King A, Silbergeld E, Morris JG (2003). Human Health Effects of Exposure to Domoic Acid in the Pacific Northwest: A Preliminary Study. Proceedings of the Behavioral Toxicology Society Conference.

[B35] Todd ECD (1993). Domoic acid and amnesic shellfish poisoning: A review. Journal of Food Protection.

[B36] Perl TM, Bedard L, Kosatsky T, Hockin JC, Todd EC, Remis RS (1990). An outbreak of toxic encephalopathy caused by eating mussels contaminated with domoic acid. New England Journal of Medicine.

[B37] Gulland FM, Haulena M, Fauquier D, Langlois G, Lander ME, Zabka T, Duerr R (2002). Domoic acid toxicity in California sea lions (*Zalophus californianus*): clinical signs, treatment and survival. Veterinary Record.

[B38] Trainer VL, Adams NG, Wekell JC, Hallegraeff GM, Blackburn SI, Bolch CJS, Lewis RJ (2001). Domoic acid-producing *Pseudo-nitzschia *species off the U.S. west coast associated with toxification events. Proceedings of the Ninth International Conference on Harmful Algal Blooms.

[B39] Thessen AE, Bowers HA, Stoecker DK, Oldach DW (2006). *Pseudo-nitzschia *spp. and domoic acid in Maryland and Virginia waters. Proceedings of the 12th International Conference on Harmful Algae; September 4–8, 2006; Copenhagen, Denmark.

[B40] Pan Y, Parsons ML, Busman M, Moeller PDR, Dortch Q, Powell CL, Doucette GJ (2001). *Pseudo-nitzschia sp. *cf. *pseudodelicatissima *– a confirmed producer of domoic acid from the northern Gulf of Mexico. Marine Ecology Progress Series.

[B41] Villareal TA, Roelke DL, Fryxell GA (1993). Occurrence of the toxic diatom *Nitzschia pungens f. multiseries *in Massachusetts Bay, Massachusetts, U.S.A. Marine Environmental Research.

[B42] Anderson DM, Lassus P, Arzul G, Erard-Le Denn E, Gentien P, Marcaillou-Le Baut C (1995). Identification of harmful algal species using molecular probes: An emerging perspective. Harmful Marine Algal Blooms.

[B43] Tillett D, Dittmann E, Erhard M, Von Dohren H, Borner T, Neilan BA (2000). Structural organization of microcystin biosynthesis in *Microcystis aeruginosa *PCC 7806: an integrated peptide-polyketide synthetase system. Chemistry and Biology.

[B44] Mihali TK, Kellman R, Muenchhoff J, Barrow KD, Neilan BA (2008). Characterization of the Gene Cluster Responsible for Cylindrospermopsin Biosynthesis. Applied and Environmental Microbiology.

[B45] Kellman R, Mihali TK, Jeon YJ, Pickford R, Pomati F, Neilan BA (2008). Biosynthetic Intermediate Analysis and Functional Homology Reveal a Saxitoxin Gene Cluster in Cyanobacteria. Applied and Environmental Microbiology.

[B46] Kubota T, Iinuma Y, Kobayashi Ji (2006). Cloning of Polyketide Synthase Genes from Amphidinolide-Producing Dinoflagellate *Amphidinium *sp. Biological and Pharmacological Bulletin.

[B47] Hackett JD, Scheetz TE, Yoon HS, Soares MB, Bonaldo MF, Casavant TL, Bhattacharya D (2005). Insights into a dinoflagellate genome through expressed sequence tag analysis. BMC Genomics.

[B48] Lidie KB, Ryan JC, Barbier M, Van Dolah FM (2005). Gene expression in Florida red tide dinoflagellate *Karenia brevis*: analysis of an expressed sequence tag library and development of DNA microarray. Marine Biotechnology.

[B49] Van Dolah FM, Lidie KB, Morey JS, Brunelle SA, Ryan JC, Monroe EA, Haynes BL (2007). Microarray analysis of diurnal- and circadian-regulated genes in the Florida red-tide dinoflagellate *Karenia brevis *(Dinophyceae). Journal of Phycology.

[B50] Trainer VL, Wekell JC, Horner RA, Hatfield CL, Stein JE, Reguera B, Blanco J, Fernández ML, Wyatt T (1998). Domoic acid production by *Pseudo-nitzschia pungens*. Harmful Algae.

[B51] Bates SS, Anderson DM, Cembella AD, Hallegraeff GM (1998). Ecophysiology and metabolism of ASP toxin production. Physiological Ecology of Harmful Algal Blooms.

[B52] Smith JC, McLachlan JL, Cormier PG, Pauley KE, Bouchard N, Smayda TJ, Shimizu Y (1991). Growth and domoic acid production and retention by *Nitzschia pungens *forma *multiseries *at low temperatures. Toxic Phytoplankton Blooms in the Sea.

[B53] Marchetti A, Trainer VL, Harrison PJ (2004). Environmental conditions and phytoplankton dynamics associated with *Pseudo-nitzschia *abundance and domoic acid in the Juan de Fuca eddy. Marine Ecology Progress Series.

[B54] Bates SS, de Freitas ASW, Milley JE, Pocklington R, Quilliam MA, Smith JC, Worms J (1991). Controls on domoic acid production by the diatom *Nitzschia pungens *f. *multiseries *in culture: nutrients and irradiance. Canadian Journal of Fisheries and Aquatic Sciences.

[B55] Pan Y, Subba Rao DV, Mann KH, Li WKW, Harrison WG (1996). Effects of silicate limitation on production of domoic acid, a neurotoxin, by the diatom *Pseudo-nitzschia multiseries *(Hasle). II. Continuous culture studies. Marine Ecology Progress Series.

[B56] Bates SS, Douglas DJ, Doucette GJ, Léger C (1995). Enhancement of domoic acid production by reintroducing bacteria to axenic cultures of the diatom *Pseudo-nitzschia multiseries*. Natural Toxins.

[B57] Kaczmarska I, Ehrman JM, Bates SS, Green DH, Leger C, Harris J (2005). Diversity and distribution of epibiotic bacteria on *Pseudo-nitzschia multiseries *(Bacillariophyceae) in culture, and comparison with those on diatoms in native seawater. Harmful Algae.

[B58] Alavi M, Miller T, Erlandson K, Schneider R, Belas R (2001). Bacterial community associated with *Pfiesteria*-like dinoflagellate cultures. Environmental Microbiology.

[B59] Jasti S, Sieracki ME, Poulton NJ, Giewat MW, Rooney-Varga JN (2005). Phylogenetic diversity and specificity of bacteria closely associated with *Alexandrium *spp. and other phytoplankton. Applied and Environmental Microbiology.

[B60] Mayali X, Doucette GJ (2002). Microbial coomunity interactions and population dynamics of an algicidal bacterium active against *Karenia brevis*. Harmful Algae.

[B61] Fisher MM, Triplett EW (1999). Automated approach for ribosomal intergenic spacer analysis of microbial diversity and its application to freshwater bacterial communities. Applied and Environmental Microbiology.

[B62] Hewson I, Fuhrman JA (2004). Richness and diversity of bacterioplankton species along an estuarine gradient in Moreton Bay, Australia. Applied and Environmental Microbiology.

[B63] Fuhrman JA, Hewson I, Schwalbach MS, Steele JA, Brown MV, Naeem S (2006). Annually recurring bacterial communities are predictable from ocean conditions. Proceedings of the National Academy of Sciences.

[B64] Sapp M, Schwaderer AS, Wiltshire KH, Hoppe H-G, Gerdts G, Wichels A (2007). Species-specific bacterial communities in the phycosphere of algae?. Microbial Ecology.

[B65] Spector DL, Spector DL (1984). Dinoflagellate Nuclei. Dinoflagellates.

[B66] Anderson DM, Lobel PS (1987). The Continuing Enigma of Ciguatera. The Biological Bulletin.

[B67] Holmes MJ, Lewis RJ, Poli MA, Gillespie NC (1991). Strain dependent production of ciguatoxin precursors (gambiertoxins) by *Gambierdiscus toxicus *(Dinophyceae) in culture. Toxicon.

[B68] Steidinger KA, Baden DG, Spector DL (1985). Toxic marine dinoflagellates. Dinoflagellates.

[B69] Kaebernick M, Neilan BA, Borner T, Dittmann E (2000). Light and the transcriptional response of the microcystin biosynthesis gene cluster. Applied Environmental Microbiology.

[B70] Lyck S, Christoffersen K (2003). Microcystin quota, cell division and microcystin net production of precultured *Microcystis aeruginosa *CYA 228 (Chroococcales, Cyanophyceae) under field conditions. Phycologia.

[B71] Orr PT, Jones GJ (1998). Relationship between microcystin production and cell division rates in nitrogen-limited *Microcystis aeruginosa *cultures. Limnology and Oceanography.

[B72] Millie DF, Fahnenstiel GL, Dyble J, Pigg R, Rediske R, Klarer DM, Litaker RW, Tester PA (2008). Influence of environmental conditions on late summer cyanobacterial abundance in Saginaw Bay, Lake Huron. Aquatic Ecosystem Health and Management.

[B73] Blackburn SI, Bolch CJS, Jones GJ, Negri A, Orr PT (1997). Cyanobacterial blooms: Why are they toxic?.

[B74] Carmichael WW (1997). The cyanotoxins. Advances in Botanical Research.

[B75] Hubbard KA, Rocap G, Armbrust EV (2008). Inter- and intra-specific community structure within the diatom genus *Pseudo-nitzschia *(Bacillariophyceae). Journal of Phycology.

[B76] Stehr CM, Connell L, Baugh KA, Bill BD, Adams NG, Trainer VL (2002). Morphological, toxicological, and genetic differences among *Pseudo-nitzschia *(Bacillariophyceae) species in inland embayments and outer coastal waters of Washington State, USA. Journal of Phycology.

[B77] McGillicuddy J, D J, Anderson DM, Lynch DR, Townsend DW (2005). Mechanisms regulating the large-scale seasonal fluctuations in *Alexandrium fundyense *populations in the Gulf of Maine: results froma physical-biological model. Deep Sea Research II.

[B78] González-Gil S, Keafer BA, Jovine RVM, Anderson DM (1998). Detection and quantification of alkaline phosphatase in single cells of phosphorus-limited marine phytoplankton. Marine Ecology Progress Series.

[B79] Adachi M, Sako Y, Ishida Y (1996). Identification of the toxic dinoflagellates *Alexandrium catenella *and *A. tamarense *(Dinophyceae) using DNA probes and whole-cell hybridization. Journal of Phycology.

[B80] Scholin CA, Buck KR, Britschgi T, Cangelosi J, Chavez FP (1996). Identification of *Pseudo-nitzschia australis *(Bacillariophyceae) using rRNA-targeted probes in whole cell and sandwich hybridization formats. Phycologia.

[B81] Casper ET, Paul JH, Smith MC, Gray M (2004). Detection and quantification of the red tide dinoflagellate *Karenia brevis *by real-time nucleic acid sequence based amplification. Applied and Environmental Microbiology.

[B82] Stevens RC, Soelberg SD, Eberhart BTL, Spencer S, Wekell JC, Chinowsky TM, Trainer VL, Furlong CE (2007). Detection of the toxin domoic acid from clam extracts using a portable surface plasmon resonance biosensor. Harmful Algae.

[B83] Dyble J, Fahnenstiel GL, Litaker RW, Millie DF, Tester PA (2008). Microcystin concentrations and genetic diversity of *Microcystis *in the lower Great Lakes. Environmental Toxicology.

[B84] Hotto A, Satchwell M, Boyer G (2005). Seasonal production and molecular characterization of microcystins in Oneida Lake, New York, USA. Environmental Toxicology.

[B85] Rinta-Kanto JM, Wilhelm SW (2006). Diversity of microcystin-producing cyanobacteria in spatially isolated regions of Lake Erie. Applied and Environmental Microbiology.

[B86] Kurmayer R, Kutzenberger T (2003). Application of real-time PCR for quantification of microcystin genotypes in a population of the toxic cyanobacterium *Microcystis *sp. Applied Environmental Microbiology.

[B87] Rinta-Kanto JM, Ouelletee AJA, Boyer GL, Twiss MR, Bridgeman TB, Wilhelm SW (2005). Quantification of toxic *Microcystis *spp. during the 2003 and 2004 blooms in western Lake Erie using quantitative real-time PCR. Environmental Science and Technology.

[B88] Kurmayer R, Dittmann E, Fastner J, Chorus I (2002). Diversity of microcystin genes within a population of the toxic cyanobacterium *Microcystis *spp. in Lake Wannsee (Berline, Germany). Microbial Ecology.

[B89] Welker M, von Dohren H, Tauscher H, Steinberg CEW, Erhard M (2003). Toxic *Microcystis *in shallow lake Muggelsee (Germany) – dynamics, distribution, diversity. Archiv fuer Hydrobiologie.

[B90] Craig SE, Lohrenz SE, Lee Z, Mahoney KL, Kirkpatrick GJ, Schofield OM, Stewrd RG (2006). Use of hyperspectral remote sensing reflectance for detection and assessment of the harmful alga, *Karenia brevis*. Applied Optics.

[B91] Scholin C, Doucette GJ, Cembella AD, Babin M, Roessler CS, Cullen JJ Prospects for developing automated systems for in situ detection of harmful algae and their toxins. Real-Time Coastal Observing Systems for Ecosystem Dynamics and Harmful Algal Blooms.

[B92] Goffredi SK, Jones W, Scholin C, Marin R, Hallam S, Vrijenhoek RC (2006). Molecular detection of marine larvae. Marine Biotechnology.

[B93] Paul J, Scholin C, Engh G van den, Perry MJ (2007). *In situ *instrumentation. Oceanography.

[B94] Falconer IR, Beresford AM, Runnegar MTC (1983). Evidence of liver damage by toxin from a bloom of the blue-green alga, *Microcystis aeruginosa*. Medical Journal of Australia.

[B95] Tester PA, Turner JT, Shea D (2000). Vectorial transport of toxins from the dinoflagellate *Gymnodinium breve *through copepods to fish. Journal of Plankton Research.

[B96] Flewelling LJ, Naar JP, Abbott JP, Baden DG, Barros NB, Bossart GD, Bottein M-YD, Hammond DG, Haubold EM, Heil CA (2005). Brevetoxicosis: Red tides and marine mammal mortalities. Nature.

[B97] Pierce RH (1986). Red tide (*Ptychodiscus brevis*) toxin aerosols: A review. Toxicon.

[B98] Pierce RH, Henry MS, Proffitt LS, Hasbrouck PA, Graneli E, Sundstrom B, Elder L, Anderson DM (1990). Red tide toxin (brevetoxin) enrichment in marine aerosol. Toxic Marine Phytoplankton.

[B99] Abraham WM, Baden DG (2006). Aerosolized Florida red tide toxins and human health effects. Oceanography.

[B100] Kirkpatrick B, Fleming LE, Backer LC, Bean JA, Tamer R, Kirkpatrick G, Kane T, Wanner A, Dalpra D, Reich A, Baden DG (2006). Environmental exposures to Florida red tides: Effects on emergency room respiratory diagnoses admissions. Harmful Algae.

[B101] Langheinrich U (2003). Zebrafish: a new model of the pharmaceutical catwalk. Bioessays.

[B102] Shin JT, Fishman MC (2002). From zebrafish to human: modular medical models. Annual Review of Genomics and Human Genetics.

[B103] Murphy TP, Irvine K, Guo J, Davies J, Murkin H, Charlton M, Watson SB (2003). New microcystin concerns in the lower Great Lakes. Water Quality Research Journal of Canada.

[B104] WHO (1998). Guidelines for Drinking Water Quality, Addendum to Volume 2, Health Criteria and Other Supporting Information.

[B105] Wilson AE, Gossiaux DC, Hook TO, Berry JP, Landrum PF, Dyble J, Guildford SJ (2008). Evaluation of the human health threat associated with the hepatotoxin microcystin, in the muscle and liver tissues of yellow perch (*Perca flavescens*). Canadian Journal of Fisheries and Aquatic Sciences.

[B106] Dyble J, Bienfang P, Dusek E, Hitchcock G, Holland F, Laws EA, Lerczak J, McGillicuddy DJ, Minnett P, Moore SK (2008). Environmental controls, oceanography and population dynamics of pathogens and harmful algal blooms: Connecting sources to human exposure. Environmental Health.

[B107] Stumpf RP, Culver ME, Tester PA, Tomlinson M, Kirkpatrick GJ, Pederson BA, Truby EW, Ransibrahmanakul V, Soracco M (2003). Monitoring *Karenia brevis *blooms in the Gulf of Mexico using satellite ocean color imagery and other data. Harmful Algae.

[B108] Reich A, Backer LC, Kirkpatrick B, Stumpf R, Fleming LE, Stephan WB, Weisman R, Jerez E, Heil C, Steidinger K (2006). Public health and Florida red tide: from remote sensing to poison information. American Public Health Association Annual Meeting; Boston, MA.

[B109] Cox PA, Banack SA, Murch SJ, Rasmussen U, Tien G, Bidigare RR, Metcalf JS, Morrison LF, Codd GA, Bergman B (2005). Diverse taxa of cyanobacteria produce b-N-methylamino-L-alanine, a neurotoxic amino acid. Proceedings of the National Academy of Sciences, USA.

[B110] Villareal TA, Moore C, Stribling P, Van Dolah F, Luber G, Wenck MA (1998). Ciguatera Fish Poisoning – Texas, and South Carolina, 2004. Morbidity and Mortality Weekly Report.

[B111] Horner RA, Garrison DL, Plumley FG (1997). Harmful algal blooms and red tide problems on the U.S. west coast. Limnology and Oceanography.

[B112] De Sylva DP (1999). Global warming and potential range extensions of poisonous and dangerous marine organisms. World Resource Review [World Resour Rev].

